# Intracellular calcium links milk stasis to lysosome-dependent cell death during early mammary gland involution

**DOI:** 10.1007/s00018-023-05044-8

**Published:** 2024-01-12

**Authors:** Jaekwang Jeong, Jongwon Lee, Gabriel Talaia, Wonnam Kim, Junho Song, Juhyeon Hong, Kwangmin Yoo, David G. Gonzalez, Diana Athonvarangkul, Jaehun Shin, Pamela Dann, Ann M. Haberman, Lark Kyun Kim, Shawn M. Ferguson, Jungmin Choi, John Wysolmerski

**Affiliations:** 1 Section of Endocrinology and Metabolism, Department of Internal Medicine, Yale University School of Medicine, New Haven, CT USA; 2 Department of Biomedical Sciences, Korea University College of Medicine, Seoul, Republic of Korea; 3 Departments of Cell Biology and of Neuroscience, Wu Tsai Institute, Yale University School of Medicine, New Haven, CT 06510 USA; 4 Division of Phamacology, School of Korean Medicine, Pusan National University, Yangsan, Gyeongnam 50612 Republic of Korea; 5 Department of Genetics, Yale School of Medicine, New Haven, CT 06510 USA; 6 Integrated Science Engineering Division, Underwood International College, Yonsei University, Seoul, Republic of Korea; 7 Departments of Immunobiology and Laboratory Medicine, Yale School of Medicine, New Haven, CT 06510 USA; 8 Department of Biomedical Sciences, Graduate School of Medical Science, Brain Korea 21 Project, Gangnam Severance Hospital, Yonsei University College of Medicine, Seoul, 06230 Republic of Korea

**Keywords:** LDCD, Calcium, PMCA2, STAT3, TFEB

## Abstract

Involution of the mammary gland after lactation is a dramatic example of coordinated cell death. Weaning causes distension of the alveolar structures due to the accumulation of milk, which, in turn, activates STAT3 and initiates a caspase-independent but lysosome-dependent cell death (LDCD) pathway. Although the importance of STAT3 and LDCD in early mammary involution is well established, it has not been entirely clear how milk stasis activates STAT3. In this report, we demonstrate that protein levels of the PMCA2 calcium pump are significantly downregulated within 2–4 h of experimental milk stasis. Reductions in PMCA2 expression correlate with an increase in cytoplasmic calcium in vivo as measured by multiphoton intravital imaging of GCaMP6f fluorescence. These events occur concomitant with the appearance of nuclear pSTAT3 expression but prior to significant activation of LDCD or its previously implicated mediators such as LIF, IL6, and TGFβ3, all of which appear to be upregulated by increased intracellular calcium. We further demonstrate that increased intracellular calcium activates STAT3 by inducing degradation of its negative regulator, SOCS3. We also observed that milk stasis, loss of PMCA2 expression and increased intracellular calcium levels activate TFEB, an important regulator of lysosome biogenesis through a process involving inhibition of CDK4/6 and cell cycle progression. In summary, these data suggest that intracellular calcium serves as an important proximal biochemical signal linking milk stasis to STAT3 activation, increased lysosomal biogenesis, and lysosome-mediated cell death.

**Supplementary Information:**

The online version contains supplementary material available at 10.1007/s00018-023-05044-8.

## Introduction

Involution of the mammary gland following lactation is one of the most dramatic examples of coordinated cell death in nature [1–4]. This process is initiated by the failure to empty milk from the gland for more than 12–24 h, which results in distension of the alveolar structures, a change in the shape of mammary epithelial cells (MECs) and programmed cell death of many epithelial cells. This first phase of involution is regulated by local mechanisms and is reversible. If the gland remains un-suckled for more than 48–72 h, a second phase of irreversible involution ensues, characterized by widespread cell death, proteolytic disruption of the basement membranes, and remodeling of epithelial and stromal components of the gland to approximate its pre-pregnant structure [1–4].

Although several pathways have been implicated in triggering the initial phase of involution [1, 4–12], a principal mediator of this process appears to be the activation of signal transducer and activator 3 (STAT3), which has been thought to occur due to the secretion of cytokines, such as leukemia inhibitory factor (LIF), interleukin 6 (IL6), and transforming growth factor (TGF) β3 by MECs in response to milk stasis [8–10, 13, 14]. STAT3, in turn, increases the number and size of lysosomes in MECs as well as the expression of lysosomal enzymes such as cathepsin B and L [8, 15, 16]. Together, these events result in a caspase-independent form of LDCD [8, 16]. Interestingly, a similar process of LDCD occurs in neurons in response to ischemia–reperfusion injury, where it is triggered, in part, by cellular calcium (Ca^2+^) overload [17, 18].

MECs transport large amounts of Ca^2+^ from the systemic circulation into milk, a process involving the plasma membrane calcium-ATPase 2 (PMCA2) [19]. PMCA2 is expressed in the apical plasma membrane of MECs specifically during lactation [19, 20] and it transports Ca^2+^ out of cells in response to ATP hydrolysis [21–23]. In its absence, milk Ca^2+^ transport is reduced by 60–70% [19, 20]. PMCA2 levels decline rapidly after weaning and PMCA2-null mice demonstrate inappropriate, widespread MEC death during lactation [20]. Given that PMCA2 is important for Ca^2+^ secretion from MECs, we hypothesized that the decline in PMCA2 levels upon weaning triggers LDCD by increasing intracellular Ca^2+^ levels. We now report that decreased PMCA2 expression is associated with increased cytoplasmic Ca^2+^ levels in MECs in vivo, and that increased intracellular Ca^2+^ triggers LIF, IL-6 and TGFβ3 expression, as well as STAT3 phosphorylation. Furthermore, we demonstrate that elevated intracellular Ca^2+^ levels activate transcriptional programs leading to lysosome biogenesis. These results suggest that an increase in intracellular Ca^2+^ due to reduced PMCA2-mediated calcium clearance represents an important proximal event coupling milk stasis to LDCD.

## Results

### Milk stasis increases intracellular calcium levels.

**Fig. 1:**
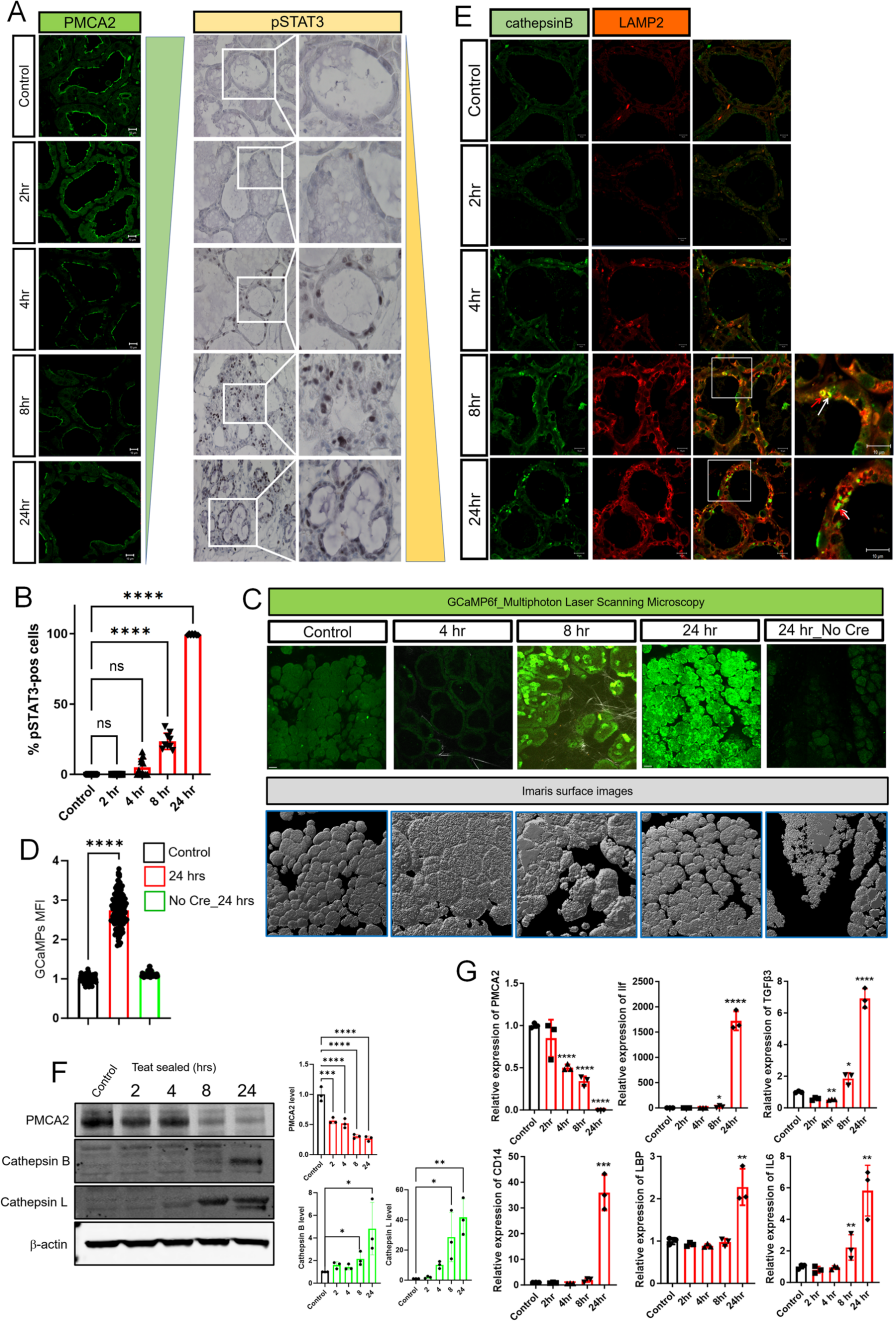
Milk stasis increases intracellular calcium levels. **A** Immunofluorescence for PMCA2 and Immunohistochemistry for pSTAT3 at 2, 4, 8, and 24 h post teat-sealing mammary gland (representative images of *n* = 3). Control represents the unsealed contralateral lactating gland. **B** Quantification of the percentage of pSTAT3-positive epithelial cells in Fig. 1. **C** Images obtained using multiphoton laser scanning microscopy at 4, 8, and 24 h post teat-sealing of a mammary gland using BLG-GCaMP6f^lox/lox^ females. The contralateral, unsealed gland served as a control. Bottom row shows matching imaris surface images (representative images of *n* = 3). **D** Mean Fluorescent Intensity (MFI) of MECs in control lactating and 24 h post teat-sealing (*n* = 3). Mice without Cre expression also served as controls. **E** Immunofluorescence for Cathepsin B and LAMP2 at 2, 4, 8, and 24 h post teat-sealing mammary gland. (representative images of *n* = 3). **F** Western blot analysis of PMCA2, Cathepsin B and Cathepsin L at 2, 4, 8, and 24 h post teat-sealing mammary gland. (*n* = 3). **G** PMCA2, Lif, TGFβ3, CD14, LBP, and IL6 mRNA expression in lactating mammary glands (control) and 2, 4, 8, and 24 h post teat-sealing, as assessed by quantitative RT-PCR (QPCR) (*n* = 3). All scale bars represent 10 μm. Bar graphs represent the mean ± SEM. * denotes *p* < 0.05, ** denotes *p* < 0.005, *** denotes *p* < 0.0005, **** denotes* p* < 0.00005

We hypothesized that a decrease in PMCA2 expression after weaning might increase intracellular Ca^2+^ levels within mammary epithelial cells and, as a result, contribute to LDCD. Experimentally, involution can be initiated in a single mouse mammary gland by sealing the teat with adhesive [24]. Given that the other 9 glands are suckled normally and continue to make milk, this model isolates the consequences of milk stasis from systemic changes caused by weaning. As shown in Fig. 1A, we confirmed that, compared to the contralateral suckled gland (control), PMCA2 immunofluorescence was significantly reduced by 4 h after teat-sealing and was decreased to very low levels by 24 h [20]. This was associated with an increase in nuclear staining for pSTAT3, which was initially detectable at 4 h after teat-sealing and progressively increased at 8 and 24 h (Fig. 1A, B).

To test whether milk stasis increased cytoplasmic Ca^2+^ levels in vivo, we generated transgenic mice expressing the genetically encoded, GCaMP6f calcium sensor specifically in MECs. GCaMP6f is a modified EGFP, which fluoresces with progressive intensity in response to increasing concentrations of cytoplasmic Ca^2+^ [25, 26]. We crossed GCaMP6f-floxed mice (formal name: Ai95(RCL-GCaMP6f)-D or Ai95 mice) to mice expressing Cre recombinase under the control of the beta-lactoglobin promoter (BLG-Cre mice) to activate expression of GCaMP6f in MECs at the transition from pregnancy to lactation [27]. Lactating BLG-Cre;GCaMP6f-floxed females underwent intravital imaging of mammary epithelial cells using multiphoton laser scanning microscopy of anesthetized mice at 4, 8, and 24 h post teat-sealing of a 4th inguinal mammary gland; the contralateral gland, which was not sealed, served as a control. We could not image individual mice sequentially for a full 24 h. Therefore, mice were either studied at 4 and 8 h, or at 24 h after teat-sealing. We used the second harmonic-generated signal from the collagen fibers within the fascia covering the glands as an anchoring point to ensure that we compared images at comparable tissue depths. As shown in Fig. 1C, D, and in Supplemental Video 1–4, teat-sealing caused a significant increase in intracellular Ca^2+^ levels in vivo, as evidenced by an increase in GCaMP fluorescence, which was first detectable at 8-h and which persisted, increased, and became more uniform at 24-h. There was no similar increase of fluorescence 24 h after teat-sealing in the glands of Ai95 females in the absence of Cre recombinase (Fig. 1C, D, Supplemental Video 5). These data demonstrate that milk stasis increases cytoplasmic Ca^2+^ levels in mammary epithelial cells in vivo.

The regulation of early involution is complex but factors implicated as important triggers of LDCD include LIF, interleukin 6 (IL-6), and transforming growth factor-beta 3 (TGFβ3), all of which are produced by MECs in response to milk stasis and can contribute to STAT3 activation [8–10, 13, 14]. In turn, STAT3 signaling has been shown to be required for LDCD in MECs after weaning [8, 28, 29]. In order to assess how temporal changes in PMCA2 expression and cytoplasmic Ca^2+^ concentration correlate with the onset of LDCD, we compared them to changes in LAMP2, cathepsin B and L, LIF, IL6 and TGFβ3 expression at 2, 4, 8, and 24 h post-teat-sealing. The contralateral gland which continued to be suckled normally served as a control in all experiments. During lactation, staining intensity for LAMP2 and cathepsin B was low but both progressively increased after teat-sealing, beginning with the 4-htime point. Immunofluorescence demonstrated an increase in the size of defined lysosomes that co-stained for both LAMP2 and Cathepsin B but also an increase in more diffuse cytoplasmic staining for both markers (Fig. 1E). At 24-h, we observed the appearance of intensely staining foci of Cathepsin B that did not co-localize with LAMP2 staining. These changes were mirrored by increasing levels of Cathepsin B and L as assessed by immunoblotting whereas PMCA2 levels declined during this same time course (Fig. 1F). We did not see any change in LIF mRNA expression until 8 h and this increase was minor compared to the prominent increase in LIF mRNA at 24-h post-teat-sealing (Fig. 1G). A similar pattern was seen for TGFβ3 and IL6 mRNA expression, and we actually noted a decrease in TGFβ3 mRNA expression at 2 and 4 h post-teat-sealing, before observing an increase in its expression over baseline at 8 and 24 h. The relative increases in IL6 and TGFβ3 mRNA levels were quantitatively much less than the increase in LIF mRNA. We also examined the expression of two STAT3-target genes that have been noted to participate in the inflammatory responses to involution, LBP (lipopolysaccharide binding protein) and CD14 (Lipopolysaccharide receptor) [7, 12]. Both mRNAs were only significantly elevated after 24 h of teat-sealing. These changes all occurred either after or concurrent with the decrease in PMCA2 expression or the increase in intracellular Ca^2+^ levels, but not before. Therefore, decreased PMCA2 expression is an early response to milk stasis, occurring before significant increases in cytoplasmic Ca^2+^ concentrations, widespread STAT3 activation or upregulation of LDCD markers. These data also demonstrate that decreased PMCA2 and increased pSTAT3 occur prior to significant increases in LIF, IL6 or TGFβ3 mRNA expression.

### Changes in PMCA2 and pSTAT3 expression are reversible with reintroduction of suckling

**Fig. 2:**
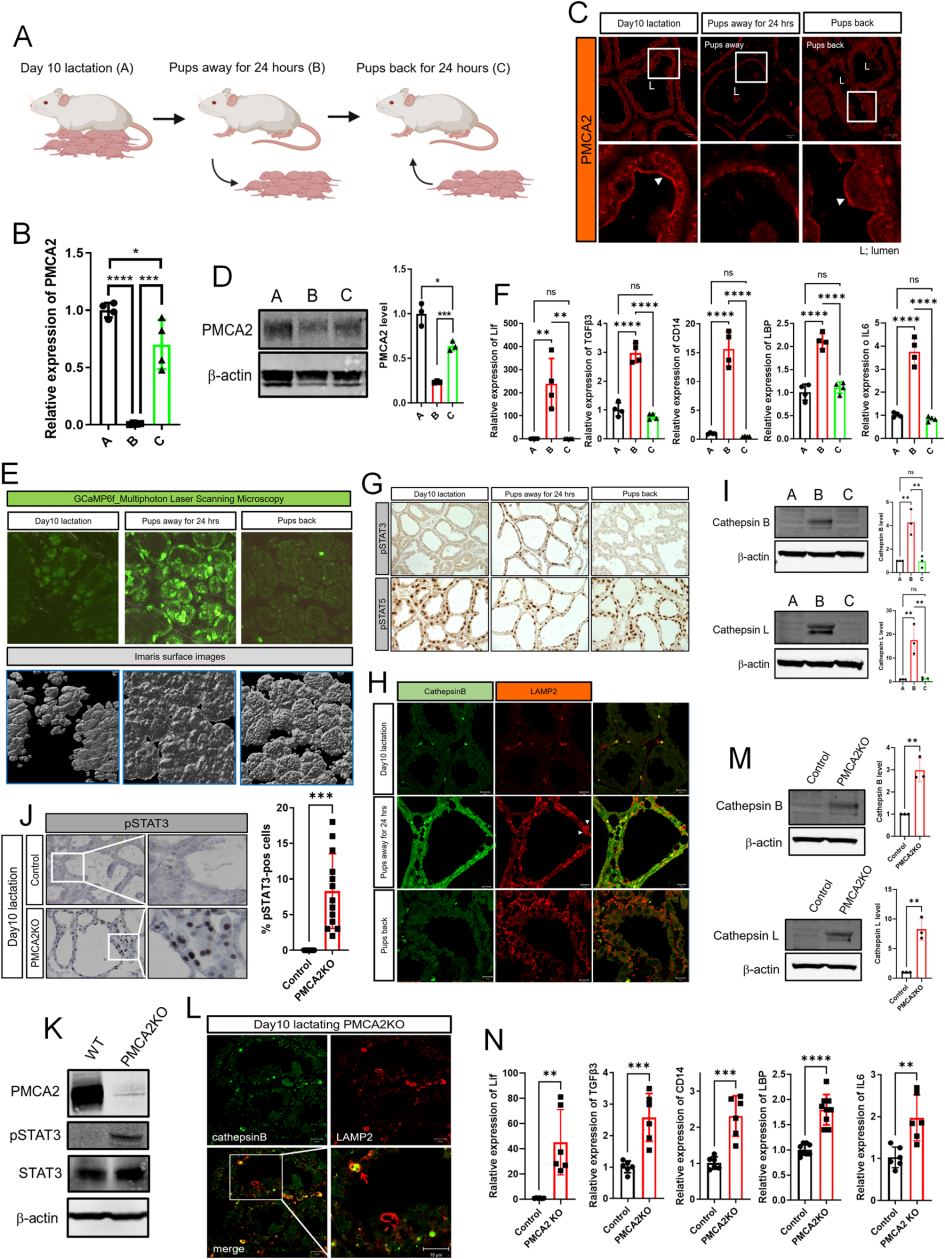
Changes in PMCA2, intracellular calcium, and pSTAT3 levels are reversible with reintroduction of suckling. **A** Experimental design. **A** Day 10 lactation as a control; **B** 24 h after pup removal at day 10 of lactation, **C** 24 h after pup reintroduction following 24-h without suckling. Created with BioRender.com. **B** PMCA2 mRNA expression, assessed by QPCR from conditions **A**–**C** (*n* = 4). **C** Immunofluorescence for PMCA2 from tissue sections of mammary glands from **A**–**C**. White arrows; apical plasma membrane (*n* = 4). **D** Western blot analysis of PMCA2 from mammary tissue extracts from **A**–**C** (*n* = 4). **E** Representative Images obtained using multiphoton laser scanning microscopy of mammary glands from conditions **A**–**C** Using BLG-GCaMP6f^lox/lox^ Bottom row shows imaris surface images (*n* = 3). **F** LIF, TGFβ3, CD14, LBP, and IL6 mRNA levels in mammary glands harvested from conditions **A**–**C**, assessed by QPCR (*n* = 4). **G** Immunohistochemistry for pSTAT3 and pSTAT5 in mammary glands harvested from conditions **A**–**C**. **H** Immunofluorescence for Cathepsin B and LAMP2 in mammary glands harvested from conditions **A**–**C**. White arrows; enlarged lysosomes. **I** Western blot analysis of Cathepsin B and Cathepsin L from tissue extracts of mammary glands harvested from conditions **A**–**C** (*n* = 4). All scale bars represent 10 μm. **J** Immunohistochemistry for pSTAT3 in mammary glands of control and PMCA2-null mice on day 10 of lactation (*n* = 3). **K** Western blot analysis of PMCA2, pSTAT3, and STAT3 in mammary glands from control and PMCA2 KO mice on day 10 of lactation (*n* = 3). **L** Immunofluorescence for Cathepsin B and LAMP2 in mammary glands from control and PMCA2 KO mice on day 10 of lactation. Red arrow; enlarged lysosome containing cathepsin B. **M** Western blot analysis for Cathepsin B and Cathepsin L in mammary gland lysates from control and PMCA2 KO mice on day 10 of lactation (*n* = 3). **N** LIF, TGFβ3, CD14, LBP, and IL6 mRNA expression assessed by QPCR in mammary glands from control and PMCA2 KO mice on day 10 of lactation (*n* = 6). All scale bars represent 10 μm. Bar graphs represent the mean ± SEM. * denotes *p* < 0.05, ** denotes *p* < 0.005, *** denotes *p* < 0.0005, **** denotes *p* < 0.00005

The first phase of mammary gland involution is reversible if pups are reintroduced to suckle within 48 h of milk stasis [1, 3, 4, 7]. We next examined whether the reversal of early involution would be associated with changes in PMCA2 expression and/or intracellular calcium. As illustrated in Fig. 2A, we compared mammary glands from three groups of lactating mice: (A) mice who were sacrificed on day 10 of lactation without manipulation; (B) mice whose pups were removed for 24 h before the mothers were sacrificed; and (C) mice whose pups were removed for 24 h and then replaced to re-suckle for 24 h before the mothers were sacrificed. As expected from the previous teat-sealing experiments, PMCA2 mRNA and protein levels were significantly reduced at 24-h after pup withdrawal (Fig. 2B–D). Remarkably, 24 h after pups were re-introduced, PMCA2 mRNA levels had recovered almost to baseline lactating levels (Fig. 2B). In addition, PMCA2 protein levels and apical membrane PMCA2 staining intensity were both increased back towards the levels noted during lactation (Fig. 2C, D). These changes in PMCA2 expression were associated with reciprocal changes in cytoplasmic Ca^2+^ as assessed by GCaMP6f fluorescence (Fig. 2E, Supplemental Video 6–7). Intracellular Ca^2+^ levels increased with weaning but were reduced back to baseline after suckling was reestablished. Like teat-sealing, pup withdrawal also led to an increase in LIF, TGFβ3, and IL6 mRNA expression, but re-suckling restored the expression of all three genes back to lactating levels (Fig. 2F). Likewise, nuclear pSTAT3 staining as well as CD14 and LBP mRNA levels were increased by pup withdrawal but were suppressed back to baseline after reintroduction of the pups (Fig. 2F, G). Interestingly, nuclear staining for pSTAT5 persisted for 24 h of pup withdrawal, at which time MECs expressed both pSTAT5 and pSTAT3 in their nuclei.

As seen in Fig. 2H, I, cathepsin B and L levels were increased at 24-h of pup withdrawal and, like pSTAT3, Cathepsin B and L levels were reduced back towards baseline 24 h after reintroduction of the pups. In contrast, LAMP2 immunofluorescence intensity was increased by pup withdrawal but remained elevated 24 h after pups were reintroduced (Fig. 2H). These data demonstrate strong correlations between PMCA2 expression, intracellular Ca^2+^ levels, activation of STAT3 and induction of LDCD markers.

### Loss of PMCA2 prematurely activates LDCD during lactation

We next examined mediators of LDCD in PMCA2-null mice in order to determine whether loss of PMCA2 was sufficient to activate this pathway. As noted previously, loss of PMCA2 expression caused premature activation of STAT3 [20] as evidenced by pSTAT3 expression in the nuclei of MECs at mid-lactation and an increase in pSTAT3 levels on immunoblots of whole mammary glands (Fig. 2J, K). Mammary glands from lactating PMCA2-null mice also demonstrated an increase in LAMP2 as well as Cathepsin B and L levels (Fig. 2L, M). Significantly, loss of PMCA2 caused an increase in LIF, TGFβ3, and IL6 mRNA levels during mid-lactation, even though continued suckling by pups removed milk, preventing milk stasis and alveolar distension (Fig. 2N). As in the teat-sealed and weaned glands (Figs. 1, 2) expression of the STAT3 targets, CD14 and LBP was also upregulated inappropriately during lactation (Fig. 2N). These data demonstrate that loss of PMCA2 is sufficient to prematurely induce cytokine expression, STAT3 activation and LDCD in MECs during active lactation, suggesting that the early decline in PMCA2 levels in response to milk stasis, serves as an important trigger for LDCD during involution.

### Increased intracellular calcium is associated with degradation of SOCS3

In order to test whether loss of PMCA2 triggers LDCD by raising intracellular Ca^2+^, we treated MCF10A cells, an immortalized but non-transformed human mammary epithelial cell line, with ionomycin and increased extracellular Ca^2+^ to increase cytoplasmic Ca^2+^ levels [30]. We examined changes in cytoplasmic Ca^2+^ by measuring the increase in fluorescence in MCF10A cells transiently transfected with a Ca^2+^ indicator, RCaMP [31]. As expected, treating MCF10A cells with 1 μM ionomycin and 10 mM extracellular Ca^2+^ for 16 h uniformly increased RCaMP fluorescence, resembling the pattern of GCaMP6f fluorescence at 24-h after teat-sealing (Supplemental Fig. 1A and Fig. 1C). It also increased nuclear pSTAT3 expression, lysosomal mass, and immunofluorescence for cathepsin B and LAMP2 (Supplemental Fig. 1 B–D). We also observed an induction of LIF, IL6 and TGFβ3 mRNA expression as well as the STAT3-target genes, CD14 and LBP (Supplemental Fig. 1E). These data demonstrate that, in MCF10A cells in vitro, increased levels of intracellular Ca^2+^ are sufficient to trigger key aspects of the STAT3-LDCD pathway.

**Fig. 3:**
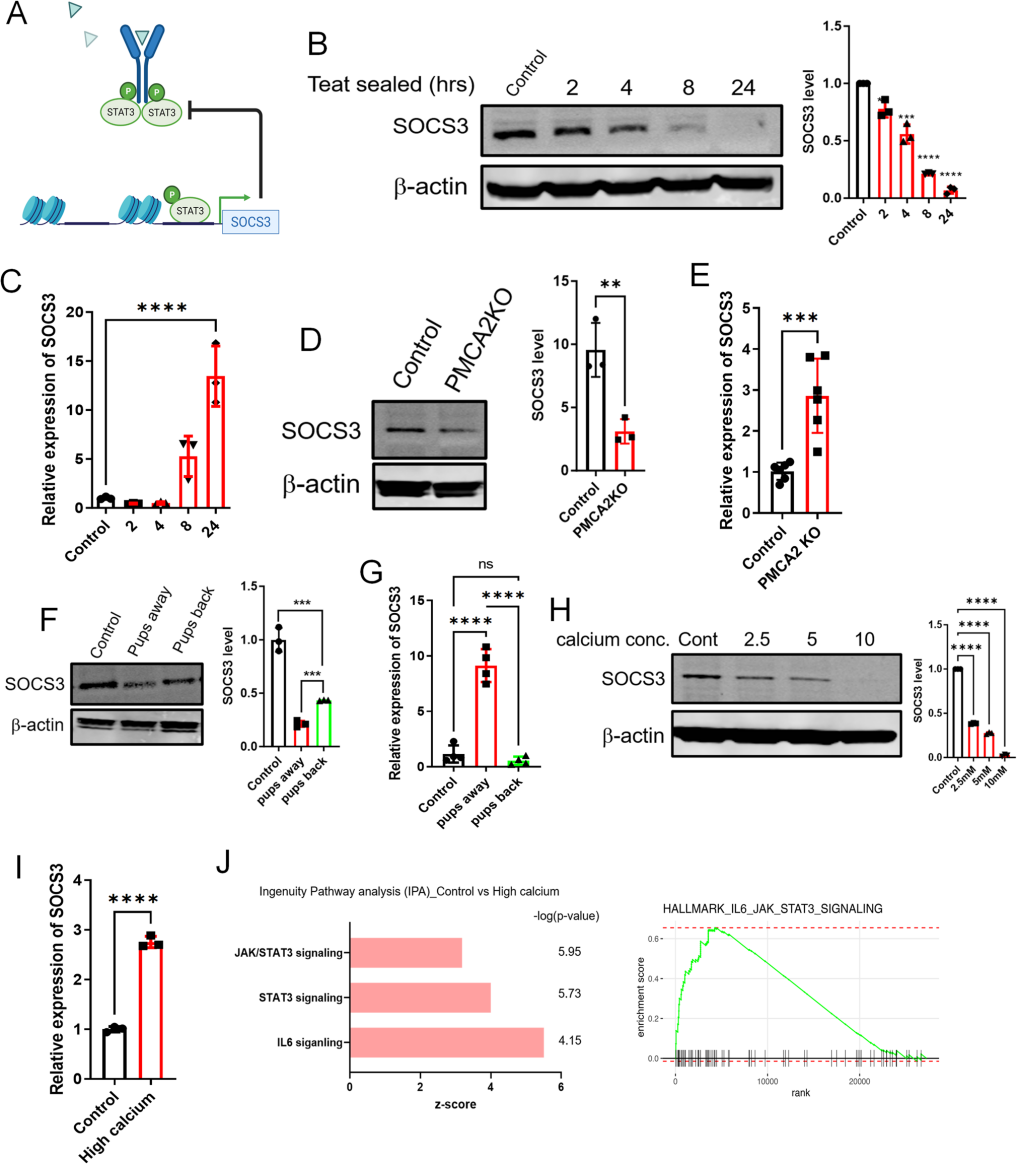
Calcium activates STAT3 signaling by reducing SOCS3 levels. **A** Diagram showing negative feedback of SOCS3 on STAT3 signaling. Created with BioRender.com. **B** Western blot analysis of SOCS3 in mammary glands harvested at 2, 4, 8, and 24 h post teat-sealing. Control is day 10 of lactation (*n* = 3). **C** Socs3 mRNA expression assessed by QPCR in mammary glands harvested at 2, 4, 8, and 24 h post teat-sealing. Control is day 10 of lactation (*n* = 3). **D** Western blot analysis of SOCS3 in mammary glands from control and PMCA2 KO mice harvested on day 10 of lactation (*n* = 3). **E** SOCS3 mRNA expression assessed by QPCR in mammary glands from control and PMCA2 KO mice harvested on day 10 of lactation (*n* = 6). **F**, **G** Western blot and QPCR analysis of SOCS3 from tissue extracts harvested from control lactating mice, mice 24 h. after teat-sealing, and mice with re-suckling of mammary glands for 24 h (*n* = 4). **H** Western blot analysis of SOCS3 in MCF10A cell exposed to increasing concentrations of extracellular calcium (control = 0, 2.5, 5, 10 mM) + 1 μM ionomycin (*n* = 3). **I** SOCS3 mRNA expression assessed by QPCR in MCF10A cells exposed to control and high calcium (10 mM calcium + 1 μM ionomycin) conditions (*n* = 3). **J** Ingenuity Pathways Analysis (IPA) for JAK-STAT3 signaling from RNAseq of MCF10A cells at control and high calcium conditions. Bar graphs represent the mean ± SEM. ** denotes *p* < 0.005, *** denotes *p* < 0.0005, **** denotes *p* < 0.00005

In MECs, STAT3 stimulates *Suppressor of Cytokine Signaling 3 *(*SOCS3*) gene expression and, in turn, SOCS3 inhibits STAT3 phosphorylation [32–34], defining a short negative feedback loop (Fig. 3A). Moreover, similar to the findings in PMCA2-null mammary glands, deletion of SOCS3 from MECs causes inappropriate activation of STAT3 during lactation [34]. Therefore, we next examined SOCS3 levels in response to teat-sealing and in PMCA2-null mammary glands. As shown in Fig. 3B, as compared to the contralateral lactating gland (time 0), SOCS3 protein levels were diminished within 2–4 h after teat-sealing and became substantially reduced by 8 and 24 h. This occurred despite a marked increase in *Socs3* mRNA levels at 8 and 24 h after teat-sealing (Fig. 3C). SOCS3 levels were also significantly reduced in lactating PMCA2-null glands as compared to wild-type lactating glands (Fig. 3D). As with teat-sealing, the pattern was the opposite for *Socs3* mRNA levels; despite the decrease in SOCS3 protein levels, there was a significant increase in *Socs3* mRNA levels in lactating PMCA2-null mammary glands as compared to wild-type control lactating glands (Fig. 3E). Similarly, reintroduction of suckling after 24-h of weaning led to reciprocal changes in SOCS3 protein and mRNA levels. Re-suckling increased SOCS3 protein (Fig. 3F) and reduced *Socs3* mRNA levels (Fig. 3G).

Given that *Socs3* gene expression is induced by STAT3, increased *Socs3* mRNA levels likely reflect the fact that SOCS3 protein is degraded in response to loss of PMCA2 expression, which we hypothesized was caused by increased intracellular Ca^2+^ [35–37]. To test this possibility, we examined the effects of increasing intracellular Ca^2+^ on SOCS3 protein and mRNA expression in MCF10A cells. As shown in Fig. 3H, treatment of these cells with ionomycin and increasing doses of extracellular Ca^2+^ resulted in a dose-dependent decrease in SOCS3 protein levels as assessed by immunoblot. Increasing intracellular Ca^2+^ levels also caused a reciprocal increase in Socs3 mRNA levels (Fig. 3I). Together, these data suggest that the loss of PMCA2 and the resulting increased levels of intracellular Ca^2+^ due to milk stasis, lead to SOCS degradation, which, in turn, increases pSTAT3 levels.

To validate these findings, we treated MCF10A cells with high extracellular calcium plus ionomycin, as described above and performed bulk RNA sequencing. As shown in Fig. 3J, treatment of MCF10A cells with calcium and ionomycin altered the expression of genes in the canonical JAK/STAT signaling pathway, in the canonical STAT3 pathway and in the canonical IL6 signaling pathway as determined by Ingenuity Pathway Analysis. Furthermore, gene set enrichment analysis (GSEA) also demonstrated upregulation of genes within the hallmark IL6-JAK-STAT3 signaling pathway.

### Increased intracellular calcium levels activate TFEB signaling to increase lysosomal biogenesis

**Fig. 4:**
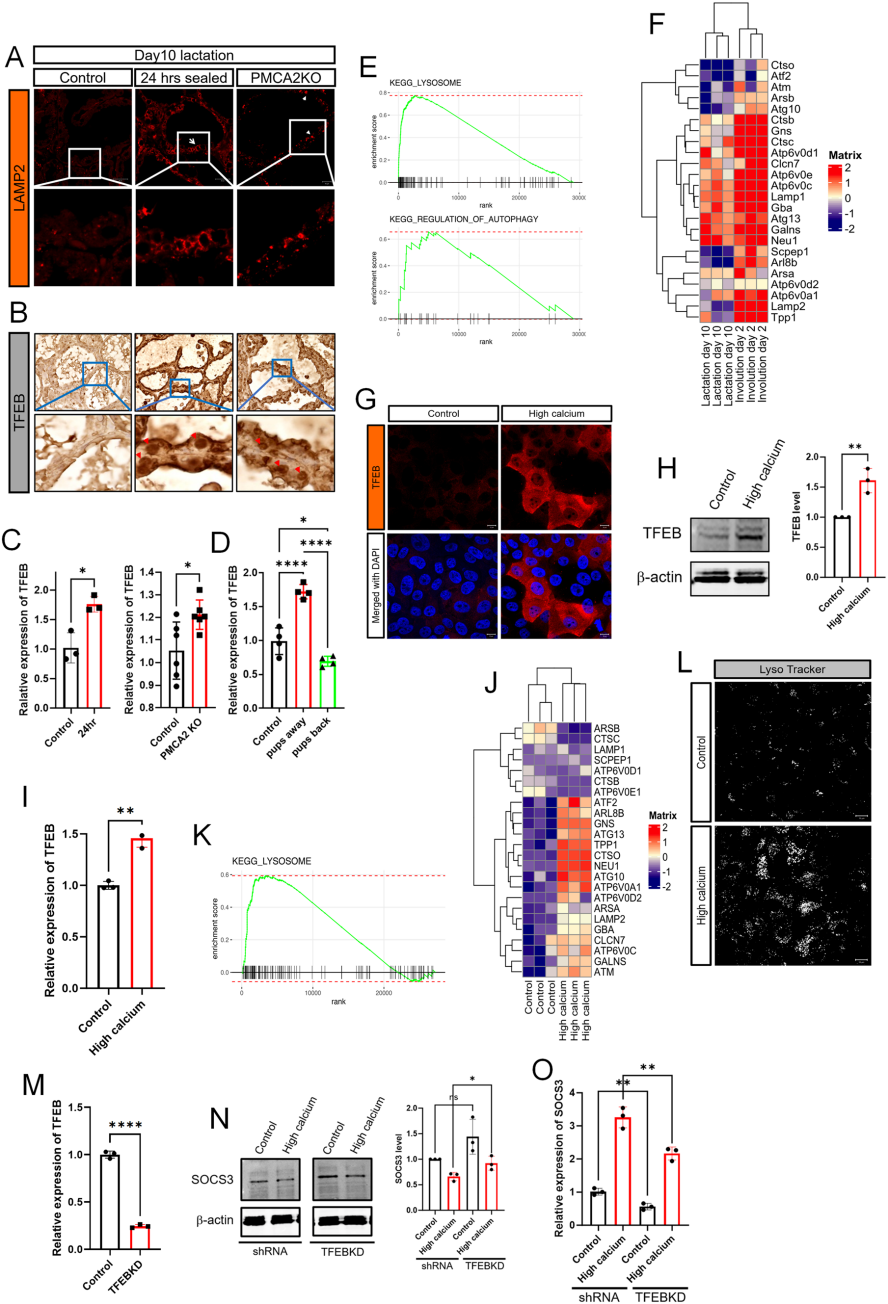
Intracellular Calcium increases TFEB and lysosome biogenesis. **A** Immunofluorescence for LAMP2 in mammary glands from control mice on day 10 of lactation, 24 h after teat-sealing, and PMCA2 KO mice on day 10 of lactation. **B** Immunohistochemistry for TFEB in mammary glands from control mice on day 10 of lactation, 24 h after teat-sealing, and PMCA2 KO mice on day 10 of lactation. **C** TFEB mRNA expression assessed by QPCR in mammary glands harvested from control mice on day 10 of lactation, 24 h after teat-sealing, and PMCA2 KO mice on day 10 of lactation (*n* = 6). **D** TFEB mRNA expression assessed by QPCR in mammary glands harvested from control lactating mice, mice 24 h after teat-sealing, and mice with re-suckling of mammary glands for 24 h (*n* = 4). **E** KEGG gene set enrichment plot of lysosome and autophagy pathway from RNAseq results compared between day 10 lactation and day 2 involution in the mammary gland. **F** Heatmap plot which highlights TFEB-regulated genes from RNAseq results compared between day 10 lactation and day 2 involution in the mammary gland. **G** Immunofluorescence for TFEB in MCF10A cells exposed to high calcium. **H** Western blot analysis of TFEB in MCF10A cells exposed to high calcium. **I** TFEB mRNA expression in MCF10A cells exposed to high calcium, assessed by QPCR (*n* = 3). **J** Heatmap plots highlight TFEB-regulated genes from RNAseq results in MCF10A cells in control versus high calcium conditions. **K** KEGG gene set enrichment plot of lysosome pathway from RNAseq results compared between control and high calcium condition in MCF10A cells. **L** Images of MCF10A cells stained by LysoTracker at control and high calcium conditions. **M** TFEB mRNA expression in control and TFEB knock down MCF10A cells (*n* = 3). **N** Western blot analysis of SOCS3 in control and TFEB knock down MCF10A cells exposed to high calcium (*n* = 3). **O** SOCS3 mRNA expression assessed by QPCR in control and TFEB knock down MCF10A cells exposed to high calcium (*n* = 3). Bar graphs represent the mean ± SEM. * denotes *p* < 0.05, ** denotes *p* < 0.005, **** denotes* p* < 0.00005

The initiation of LDCD involves an increase in lysosome mass as well as increases in lysosome membrane permeability [8, 16]. Consistent with this observation, we found expression of the lysosomal marker, LAMP2, to be increased in response to teat-sealing, pup withdrawal and in lactating PMCA2-null glands (Figs. 1, 2, Supplemental Fig. 1 and Fig. 4A). An important regulator of lysosome biogenesis is the transcription factor EB (TFEB) [38, 39] and immunostaining for TFEB revealed increased total and nuclear staining in both the teat-sealed mammary gland and in lactating PMCA2-null glands as compared to lactating control gland, which did not show any nuclear TFEB staining and had less cytoplasmic staining as well (Fig. 4B). TFEB mRNA levels were also increased in response to teat-sealing and in PMCA2-null glands during lactation (Fig. 4C). Likewise, withdrawal of pups for 24 h increased TFEB mRNA levels, but reintroduction of pups after 24 h of weaning, led to a reduction of TFEB mRNA levels back to below baseline levels, correlating with changes in PMCA2 expression and intracellular Ca^2+^ levels as noted previously (Figs. 2, 4D). We also detected changes in gene expression consistent with activation of TFEB when we interrogated a RNAseq database which included mouse mammary glands harvested at day 10 of lactation and at day 2 of involution [40]. KEGG-identified GSEA demonstrated an increase in the expression of genes associated with lysosomes and autophagy at day 2 of involution as compared to lactation, both of which are processes known to be regulated by TFEB (Fig. 4E). Furthermore, involution was associated with an increase in the expression of genes previously identified as specifically regulated by TFEB, including multiple lysosomal proteins, such as the cathepsin and Lamp families (Fig. 4F).

These results in vivo were reinforced by results in mammary epithelial cells in vitro. As shown in Fig. 4G, incubating MCF10A cells with 1 μM ionomycin and 10 mM extracellular Ca^2+^ for 16 h led to a dramatic increase in immunofluorescence for TFEB, both in the cytoplasm and in nuclei. This was associated with an increase in TFEB protein levels by immunoblot and TFEB mRNA levels as measured by QPCR (Fig. 4H,I). In addition, analysis of RNAseq data from MCF10A cells treated with high calcium demonstrated activation of genes known to be regulated by TFEB and KEGG-identified GSEA demonstrated a significant increase in the expression of genes associated with lysosomes (Fig. 4J, K). LysoTracker staining confirmed the increase of lysosomal mass in MCF10A cells treated with high calcium (Fig. 4L). Finally, we used TFEB-knockdown MCF10A cells to determine whether the induction of TFEB might influence the ability of high calcium to affect SOCS3 levels (Fig. 4M). As shown in Fig. 4N, O, knocking down TFEB resulted in a tendancy towards higher baseline levels of SOCS3 protein but had minimal effects on the ability of elevated intracellular calcium to reduce SOCS3 protein levels. However, this did result in a modest reduction of SOCS3 mRNA levels in the setting of high calcium.

### Activation of TFEB signaling by elevations in intracellular calcium is associated with alterations in cell cycle regulatory factors

Nuclear translocation of TFEB is controlled by multiple mechanisms, including by calcineurin signaling, by mTOR signaling and by the cell cycle regulators, CDK4/6 [41, 42]. Increasing intracellular calcium levels in MCF10A cells activated the GCaMP3-TRPML1 calcium sensor, demonstrating an increase in lysosomal Ca^2+^ content and export [43] (Supplemental Fig. 2A). As expected, this was also associated with nuclear translocation of NFAT, a standard bioassay of calcineurin activity [44] (Supplemental Fig. 2B). However, treatment of MCF10A cells with the calcineurin inhibitor, cyclosporin A, did not prevent the increase in lysosomal mass as determined by lysotracker staining (Supplemental Fig. 2C), suggesting that calcineurin signaling was not a dominant pathway leading to increased TFEB activity in these cells. We next examined whether increased intracellular calcium inhibited mTOR signaling, which has also been reported to regulate nuclear accumulation of TFEB [45, 46]. However, treatment with calcium and ionomycin either promoted or did not affect mTOR activity as determined by the phosphorylation of 3 mTOR targets, S6, S6K1, and ULK1 (Supplemental Fig. 2D). Thus, neither activation of calcineurin activity nor inhibition of mTOR appeared to explain the increase in total and nuclear TFEB in response to calcium/ionomycin.

**Fig. 5:**
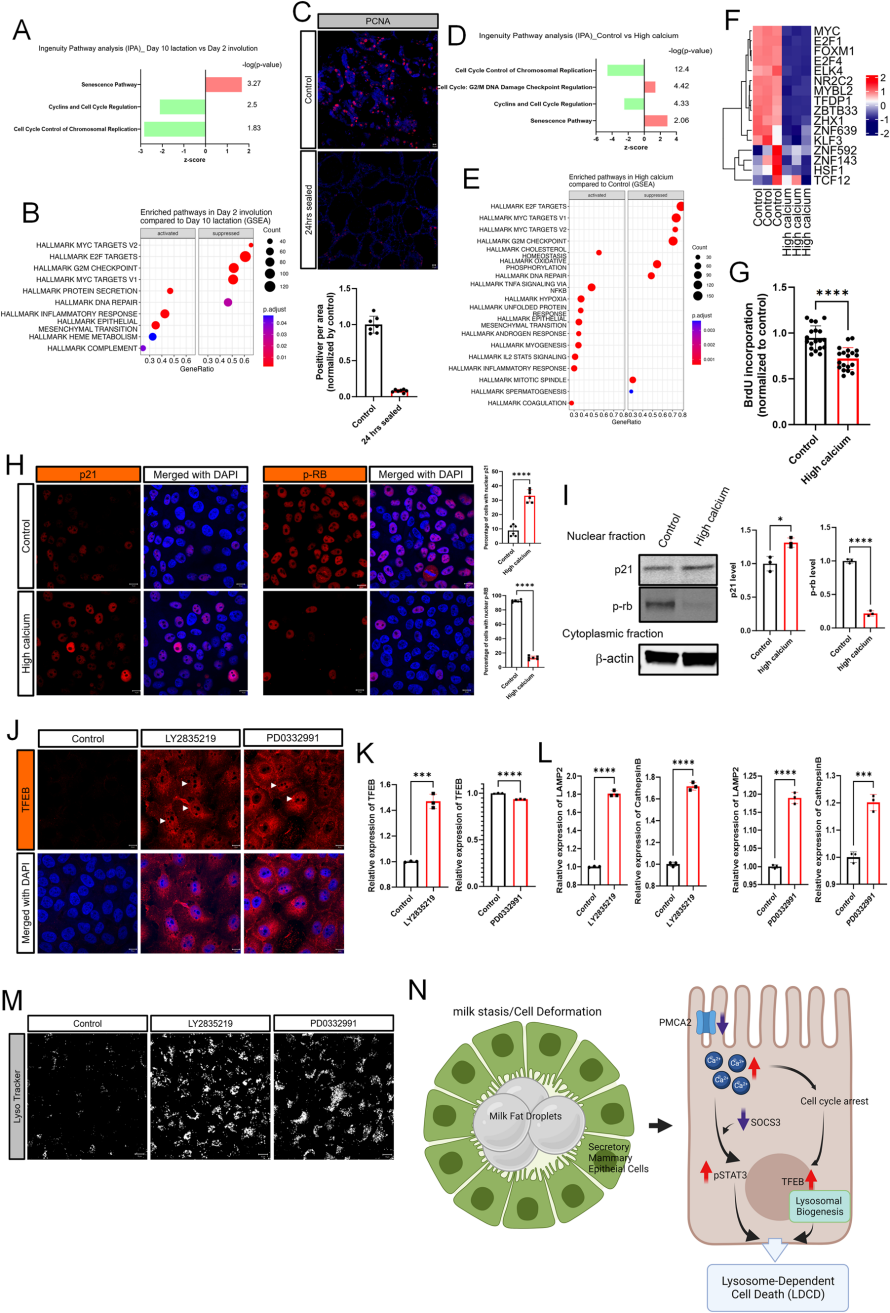
Intracellular calcium increases TFEB through inhibition of cell cycle progression. **A**, **B** Ingenuity Pathways Analysis (IPA) and GSEA highlighting cell cycle related pathways from RNAseq results comparing day 10 lactation with day 2 involution in the mammary gland. **C** Immunofluorescence of PCNA in day 10 lactation and day 1 involution of mammary gland D&E) Ingenuity Pathways Analysis (IPA) and GSEA highlighting cell cycle related pathways from RNAseq results comparing control and high calcium treated MCF10A cells. **F** Heatmap plots highlighting cell cycle-regulated genes from RNAseq results in MCF10A cells in control versus high calcium conditions. **G** Measurement of BrdU incorporation in MCF10A cells at control versus high calcium conditions. **H** Immunofluorescence for p21 and p-RB under high calcium conditions. **I** Western blot analysis of nuclear p21 and p-RB in MCF10A cells under high calcium conditions (*n* = 3). **J** Immunofluorescence for TFEB in MCF10A cells exposed to 2.5 μM LY2835219 or 5 μM PD0332991. **K** TFEB mRNA expression assessed by QPCR in MCF10A cells exposed to LY2835219 or PD0332991 (*n* = 3). **L** LAMP2 and Cathepsin B mRNA expression assessed by QPCR in MCF10A cells exposed to LY2835219 or PD0332991 (*n* = 3). **M** LysoTracker staining of MCF10A cells at baseline, and after treatment with LY2835219 or with PD0332991. All scale bars represent 10 μm. **N** Working model illustrating how milk stasis initiates LDCD by decreasing PMCA2 levels and increasing intracellular Ca^2+^. Created with BioRender.com. Bar graphs represent the mean ± SEM. * denotes *p* < 0.05, *** denotes *p* < 0.0005, **** denotes *p* < 0.00005

We next focused on the possibility that alterations in cell cycle regulation might affect TFEB expression and/or nuclear translocation given that CDK4/6 have been shown to phosphorylate TFEB, inhibiting its nuclear localization [42]. In support of this possibility, ingenuity pathway analysis and gene set enrichment analysis (GSEA) of the previously described mammary gland RNAseq data comparing lactating and 48 h of involution showed that involution was associated with upregulation of the “senescence” pathway and downregulation of the “cyclin and cell cycle regulation” and “cell cycle control of chromosomal replication” pathways (Fig. 5A). In addition, GSEA showed significant decreases in genes involved in the following KEGG pathways: cell cycle, DNA replication, E2F targets, DNA repair and MYC targets (Fig. 5B and Supplemental Fig. 3A). These data were consistent with a reduction of proliferation in teat-sealed gland at 24-h as compared to 12 days of lactation (Fig. 5C).

RNAseq data from MCF10A cells treated with calcium and ionomycin showed similar results. Ingenuity Pathway analysis demonstrated an increase in the “cell cycle: G2/M DNA damage checkpoint regulation” and “senescence” pathways along with a decrease in “cell cycle control of chromosomal replication” and “cyclins and cell cycle regulation” pathways (Fig. 5D). Gene set enrichment analyses demonstrated a decrease in the expression of genes involved in KEGG hallmark pathways for cell cycle, DNA replication, DNA repair and MYC targets (Supplemental Fig. 3B and Fig. 5F). RNAseq based DoRothEA (Discriminant Regulon Expression Analysis) analysis confirmed that the activity of transcription factors involved in cell cycle progression was downregulated in MCF10A cells treated with high calcium (Fig. 5F). Finally, these changes were supported by the reduction in BrdU incorporation of MCF10A cells treated with calcium and ionomycin (Fig. 5G). These changes support the idea that mammary gland involution is associated with inhibition of cell cycle progression.

CDK4/6 activity can be inhibited by increased levels of p21, resulting in hypophosphorylation of retinoblastoma protein Rb [47–49]. We therefore examined p21 expression and phosphorylation of Rb in MCF10A cells treated with calcium/ionomycin and saw an increase in the proportion of cells with nuclear staining for p21 and a decrease in the proportion of cells staining for nuclear pRb (Fig. 5H). We confirmed these immunofluorescence patterns by also performing immunoblots for p21 and pRb in nuclear extracts from MCF10A cells treated with calcium/ionomycin. As shown, calcium/ionomycin increased p21 levels and decreased pRb levels in nuclear extracts of MCF10A cells (Fig. 5I). Finally, we used two CDK4/6 inhibitors, LY2835219 (Abemaciclib) and PD0332991 (Palbociclib), both of which are used in breast cancer patients, to see whether inhibition of CDK4/6 would reproduce the effects of calcium/ionomycin on TFEB activity. Treatment of MCF10A cells with either PD0332991 or LY2835219 led to an increase in total and nuclear immunofluorescence for TFEB (Fig. 5J). Interestingly, LY2835219, but not PD0332991, also increased *TFEB gene* expression (Fig. 5K). Despite this difference, gene expression was increased for LAMP2 and Cathepsin B, both known targets of TFEB (Fig. 5L). Both treatments also increased lysosomal mass (Fig. 5M). Taken together, these data suggest that, during early involution, increased intracellular calcium inhibits CDK4/6 activity, which, in turn, increases TFEB expression, nuclear localization, and signaling to increase lysosome biogenesis, part of a coordinated LDCD pathway.

## Discussion

The coordinated death of MECs upon weaning is an important feature of mammalian reproduction that allows for the cyclical production of milk following multiple pregnancies, while avoiding the energetic burden of maintaining constant milk production between pregnancies [1, 3, 4, 7]. Therefore, it is important to better understand the molecular mechanisms that underlie this process. A series of studies have provided the following working model of early involution: alveolar distension due to milk retention increases cytokine production and activates STAT3, which, in turn, triggers lysosome-mediated cell death pathways [2, 7, 8, 10, 13, 14, 16, 24, 28, 29]. However, the mechanisms by which milk stasis activates cytokine production and STAT3 phosphorylation have been less clear. We now present evidence demonstrating that milk stasis rapidly decreases the expression of the calcium pump, PMCA2, causing a sustained increase in intracellular Ca^2+^ concentration. This rise in intracellular Ca^2+^ activates LIF, TGFβ3, and IL-6 production, inhibits cell cycle progression, triggers SOCS3 degradation, activates STAT3 signaling, and activates TFEB signaling, all of which contribute to the initiation of LDCD. Interestingly, the fall in PMCA2 levels as well as the increase in intracellular Ca^2+^ generally precede, and are sufficient for the increase in LIF, TGFβ3 and IL6 expression, all cytokines that have previously been suggested to mediate the effects of milk stasis on cell death [8–10, 13, 14]. In addition, increased cellular Ca^2+^ levels correlate with an inhibition in cell proliferation and increased TFEB signaling. Our observations suggest a working model (Fig. 5N), whereby the rise in intracellular Ca^2+^ acts as an early biochemical signal for initial activation of STAT3. Increased intracellular calcium also increases local cytokine production, TFEB activation and lysosome biogenesis leading to an amplification of STAT3 activation, and an increase in lysosomal mass, both of which support the full development of LDCD.

Our results suggest that increased intracellular Ca^2+^ activates STAT3 by decreasing SOCS3 levels. The decrease in SOCS3 protein occurs despite a reciprocal increase in SOCS3 mRNA levels, demonstrating that increased Ca^2+^ triggers degradation of SOCS3 protein, disinhibiting STAT3-induced expression of the *Socs3* gene. Sutherland and colleagues had previously reported that mammary-specific disruption of the *Socs3* gene led to premature STAT3 phosphorylation and cell death during lactation, mirroring the phenotype of lactating PMCA2-null mice [33, 34]. Furthermore, the authors demonstrated an increase in *Socs3* mRNA in control animals 24 h after weaning, findings consistent with our data. It is of interest that SOCS3 has previously been shown to interact with cell cycle regulators but it is thought to contribute to cell cycle arrest by magnifying p53-mediated upregulation of the cyclin-dependent kinase inhibitor, CDKN1A or p21 [50, 51]. Our observations of decreased levels of SOCS3 in the setting of increased p21 levels do not fit this model; therefore, further work will be required to understand whether decreases in SOCS3 protein levels in mammary epithelial cells are mediated by shared pathways involving cell cycle regulation that also increase TFEB activation.

Our data demonstrate that increased intracellular calcium is associated with a decrease in SOCS3 levels in vitro, and our time course after teat-sealing demonstrates a decrease in SOCS3 levels as early as 4 h after teat-sealing. However, while immunohistochemistry suggested an initial increase in pSTAT3-positive cells at 4-h, the change in pSTAT3 was variable and was not statistically significant when we counted the percentage of pSTAT3-positive cells at this time point. These changes in SOCS3 and pSTAT3 occur prior to detecting an increase in GCaMP6f fluorescence at 8 h. We suspect that immunohistochemistry and immunoblotting for changes in pSTAT3 and SOCS3 may be more sensitive than our ability to detect changes in GCaMP6f calcium sensor activation, especially since PMCA2 levels decline by 2 h. However, it is also possible that the initial decline in SOCS3 may be triggered by regional increases in intracellular calcium near the plasma membrane, as happens in breast cancer cells when PMCA2 levels are decreased [52, 53]. Alternatively, the very initial activation of STAT3 may be related to other changes brought about by early decreases in PMCA2 expression. Either way, it is clear that, sometimes between 4 and 8 h after teat-sealing, intracellular calcium rises throughout the cytoplasm and this contributes to decreases in SOCS3, activation of STAT3, and initiation of LDCD.

Activation of the LDCD pathway during early mammary gland involution is associated with an increase in the number and size of lysosomes. Lysosome biogenesis is coordinated by several transcription factors, including TFEB, which upregulates a network of genes encoding proteins important to the structure and function of lysosomes [38, 39, 54]. We found that milk stasis and loss of PMCA2 expression both upregulate TFEB expression and nuclear localization in vivo. This was associated with an increase in TFEB-regulated genes [40]. We also demonstrated that increased intracellular Ca^2+^ increases TFEB expression and nuclear localization in mammary epithelial cells in vitro and this was also associated with an increase in the expression of TFEB-regulated genes based on RNAseq data. Furthermore, our data demonstrate a clear reciprocal relationship between activation of TFEB pathways and inhibition of cell cycle progression based on RNAseq data in involuting mammary glands in vivo and in MCF10A cells treated with calcium/ionomycin. These data suggest that the inhibition of cell cycle progression may mediate the effects of intracellular calcium on TFEB expression and nuclear localization. Inhibition of cell cycle progression has previously been shown to promote nuclear localization of TFEB and activation of TFEB-associated gene expression [41, 42]. It has been shown that CDK4/6 can directly phosphorylate TFEB leading to its nuclear exclusion and degradation [41], findings consistent with our observations that pharmacologic inhibition of cell cycle progression using two different CDK4/6 inhibitors increases total and nuclear TFEB levels, increases the expression of downstream TFEB target genes and increases lysosomal mass. Thus, we propose that intracellular calcium increases TFEB signaling by inhibiting CDK-mediated cell cycle progression.

Although not all secretory epithelial cells die during the first phase of involution, it appears that the majority demonstrate increased GCaMP6f fluorescence by 24.h after teat-sealing (Fig. 1C). Interestingly, there is more heterogeneity in GCaMP6f fluorescence 24.h after weaning as suggested in Fig. 2E. This may suggest variability in either the degree of intracellular calcium overload and/or the response to elevated calcium among epithelial cells. It is interesting to speculate whether this might dictate which cells survive the first phase of involution. However, it is difficult to co-register calcium accumulation in vivo with activation of LDCD using intravital calcium imaging and our models in vitro demonstrate uniform elevations in intracellular calcium (Supplemental Fig. 1A). Further work will be needed to address potential heterogeneity in these responses.

In summary, we present data identifying intracellular calcium as an important early signal linking milk stasis to mammary epithelial cell death. We found that milk retention causes a decrease in PMCA2 expression and a rise in intracellular Ca^2+^. The increase in intracellular Ca^2+^, in turn, activates STAT3 by degrading SOCS3. Elevated intracellular calcium also increases TFEB expression, nuclear localization, and signaling leading to lysosome expansion after weaning. We propose that these events function as key proximal signals initiating and amplifying LDCD in MECs during early involution, a process that triggers the death of secretory MECs and, ultimately, initiates the preparation of the mammary gland for a new cycle of reproduction.

## Materials and methods

### Cell culture

MCF10A cells were grown in 2 dimensional monolayer on plastic, and were cultured in DMEM/F12 (Gibco-Life Technologies) containing 5% horse serum, EGF (100 ng/ml), hydrocortisone (1 mg/ml), cholera toxin (1 mg/ml), insulin (10 μg/ml), and pen/strep (Gibco-Life Technologies) at 37 °C in 5% CO_2_ [55]. In some experiment, cells were cultured under high calcium conditions (10 mM calcium + 1 μM ionomycin) for 16 h. To inhibit cell cycle progression, cells were treated with LY2835219 (2.5 μM) and PD0332991 (5 μM) (Selleckchem, Randnor, PA) for 16 h.

### Genetically-altered mice

PMCA2wt/dfw-2J mice were obtained from Jackson Laboratory (CByJ.A-Atp2b2dfw-2J/J, stock number 002894). Ai95(RCL-GCaMP6f)-D (Ai95) were a gift of the Lawrence Cohen laboratory at Yale University and were crossed with BLG-Cre mice, which were the gift of the Christine Watson Laboratory at the University of Cambridge. All animal experiments were approved by the Yale Institutional Animal Care and Use Committee.

### Immunofluorescence

Cells were grown on coverslips, fixed in 4% paraformaldehyde for 20 min, permeabilized with 0.2% Triton X100 for 10 min, washed three times with PBS and incubated with primary antibody overnight at 4 °C. The cells were washed three times with PBS and incubated with secondary antibody for 1 h at room temperature. After washing, coverslips were mounted using Prolong Gold antifade reagent with DAPI (Invitrogen). Paraffin-embedded tissue sections were cleared with histoclear (National Diagnostics) and graded alcohol using standard techniques. Antigen retrieval was performed using 7 mM citrate buffer, pH 6.0 under pressure. Sections were incubated with primary antibody overnight at 4 °C and with secondary antibody for 1 h at room temperature. Coverslips were mounted using Prolong Gold antifade reagent with DAPI (Invitrogen)***.*** All images were obtained using a Zeiss 780 confocal microscope and Zeiss LSM 880, and settings were adjusted to allow for detection of fine membrane structure. Primary antibodies were against: LAMP2 (ab13524) from Abcam (Cambridge, MA); PMCA2 (PA1-915) from Thermo Scientific (Waltham, MA); cathepsin B (PA5-17007) and TFEB (PA5-96632) from Invitrogen (Grand Island, NY); Phospho-STAT3 (9145), p21 (2947), p-Rb (8516), and NFAT (5861) from cell signaling (Danvers, MA); PCNA (sc-25280) from Santa Cruz (Dallas, TX).

### Immunohistochemistry

Paraffin-embedded tissue sections were cleared with histoclear (National Diagnostics) and graded alcohol using standard techniques. Immunohistochemistry was performed using standard techniques [56]. Antigen retrieval was accomplished by heating sections in 7 mM or 10 mM citrate, under pressure. Sections were incubated with primary antibody overnight at 4 °C. Staining was detected using Vector Elite ABC Kits (Vector Laboratories, Burlingame, CA, USA) and 3,3-diaminobenzidine as chromogen (Vector Laboratories). Primary antibodies were against: phospho-STAT3 (9145), phospho-STAT5 (9314), CREB (9197), phospho-CREB (9198), and NFAT (5861), all from cell signaling (Danvers, MA) as well as TFEB (PA5-96632) from Invitrogen (Grand Island, NY).

### Intravital multiphoton microscopy

Mice were initially anaesthetized with an intraperitoneal injection of ketamine (15 mg/mL) and xylazine (1 mg/mL) in PBS and maintained throughout the course of the experiment with vaporized isoflurane, 1.5% in oxygen, on a heating pad maintaining temperature at 37 °C. The abdomen was shaved using a mechanical trimmer and depilatory cream and the inguinal mammary gland was surgically exposed on a skin flap. The surrounding tissue was pinned to a silicone mount to stabilize and prewarmed PBS (37 °C) was applied topically to the flap throughout the imaging procedure. A coverslip mounted on a micromanipulator was lowered onto the mammary gland prior to imaging.

Image stacks were acquired with a LaVision TriM Scope II (LaVision Biotec) microscope equipped with a Chameleon Vision II (Coherent) multiphoton laser. The laser was tuned to 880 nm, focused through a × 20 water immersion lens (N.A. 1.0; Olympus) and scanned a field of view of 0.5 mm^2^ at 800 Hz (0.48 µm/pixel). Serial optical sections were acquired in 3-μm steps to image a total depth of ∼70 μm of tissue. Larger regions were visualized using a motorized stage to automatically acquire sequential fields of view in a 3 × 3 grid with 4% overlap between regions. Laser power and imaging settings was consistently maintained between all replicates. Emitted fluorescence was collected through two non-descanned detectors and separated through a dichroic (490 nm) and bandpass filters (435/90 = blue, 525/50 = green).

Image stacks were initially stitched by a grid/collection stitching plugin in Fiji before importing into Imaris software v9.2.1 (Bitplane) for three-dimensional volume rendering. Surfaces were created based on the green-fluorescent signal and manually segmented into individual alveoli for analysis of their mean fluorescent intensity.

In vivo results, represent samples from three individual mice for teat-sealing experiments and two mice for the pup withdrawal and reintroduction experiments were used. An unpaired Student'’s *t* test was used for all analyses with a *P* value of less than 0.05 accepted as indicating a significant difference. Statistical calculations were performed using the Prism (GraphPad).

### Immunoblotting

Protein extracts were prepared using standard methods [55, 57], subjected to SDS-PAGE and transferred to a nitrocellulose membrane by wet western blot transfer (Bio-Rad). The membrane was blocked in TBST buffer (TBS + 1% Tween) containing 5% milk for 1 h at room temperature. The blocked membranes were incubated overnight at 4 °C with primary antibodies in Odyssey blocking buffer, 927–40,000, washed three times with TBST buffer, and then incubated with secondary antibodies provided by LI-COR for 2 h at room temperature. After three washes with TBST buffer, the membranes were analyzed using the ODYSSEY Infrared Imaging system (LI-COR). Primary antibodies were against: PMCA2 (PA1-915) from Thermo Scientific (Waltham, MA); phospho-STAT3 (9145), STAT3 (9139), p21 (2947), p-Rb (8516), SOCS3 (2923), Phospho-S6 Ribosomal Protein (Ser235/236) (4858), S6 Ribosomal Protein (5G10) (2217), Phospho-p70 S6 Kinase (Thr389) (9205), p70 S6 Kinase Antibody (9202), Phospho-ULK1 (Ser757) (D7O6U) (14202), ULK1 (D8H5) (8054)), cathepsin B (31718), and cathepsin L (71298) from cell signaling (Danvers, MA); SOCS3 (HPA068569) from sigma (Burlington, MA); mouse (sc-69879), and rabbit (sc-130656) β-actin from Santa Cruz (Dallas, TX); TFEB (PA5-96632), and cathepsin B (PA5-17007) from Invitrogen (Grand Island, NY); SOCS3 (ab16030) from Abcam (Cambridge, MA). All immunoblot experiments were performed at least three times and representative blots are shown in the figures.

### Knockdown cell line

A stable cell line expressing shRNA directed against TFEB was generated by transducing cells with commercially prepared lentiviruses containing three individual shRNA directed against TGFBR2 (sc-36657-v) and TFEB (sc-38509-v) mRNA (Santa Cruz). Cells were cultured in 6-well plates and infected by adding the shRNA lentiviral particles to the culture for 48 h per the manufacturer’s instructions. Stable clones expressing the specific shRNAs were selected using 5 μg/ml of puromycin (Gibco-life technologies) and pooled to generate the cells used in the experiments.

### Cell transfections

Constructs encoding pCAG cyto-RCaMP1h (plasmid #105014), and GcaMP3-TRPML were a gift of Haoxing Xu in the University of Michigan. Cells were transfected using Fugene6 transfection reagent (Invitrogen) according to the manufacturer’s instructions.

### RNA extraction and real-time RT-PCR

RNA was isolated using TRIzol (Invitrogen). Quantitative RT-PCR was performed with the SuperScript III Platinum One-Step qRT-PCR Kit (Invitrogen) using a Step One Plus Real-Time PCR System (Applied Biosystems) and the following TaqMan primer sets: human and mouse Lif (Hs01055668_m1 and Mm00434761_m1), mouse PMCA2 (Mm00437640_m1), human and mouse CD14 (Hs02621496_s1 and Mm00438094_g1), human and mouse LBP (Hs01084628_m1 and Mm00493139_m1), human and mouse IL6 (Hs00174131_m1 and Mm00446190_m1), human and mouse SOCS3 (Hs02330328_s1 and Mm00545913_s1), human and mouse TGFβ3 (Hs01086000_m1 and Mm00436960_m1), human LAMP2 (Hs00174474_m1), and human CTSB (Hs00947439_m1), and human and mouse TFEB (Hs00292981_m1 and Mm00448968_m1). Human HPRT1 (4325801) and mouse GAPD (4351309) were used as reference genes (Invitrogen). Relative mRNA expression was determined using the Step One Software v2.2.2 (Applied Biosystems).

### Bulk RNA sequencing

RNA sequencing was performed by the Yale Center for Genome Analysis using the Illumina NovaSeq 6000 system, with 2 × 100 bp paired end. The sequencing reads were aligned onto the mouse GRCm38/mm10 and the Human GRCh38/hg38 reference genomes using the HISAT2 ver.2.1.0 [58] software. The mapped reads were converted into the count matrix using StringTie2 ver. 2.1.4 [59] with the default parameters, and provided to DESeq2 ver. 1.32.0 [60] to identify differentially expressed genes (DEGs) based on a negative binomial generalized linear models. Genes that satisfy |Log2 Fold Change|≥ 0.25 and adjusted *p* values < 0.05 were considered as statistically significant. The data visualization of the DEGs along with TFEB related genes and hierarchical clustered heatmaps were performed using the EnhancedHeatmap package [61] in R.

### Gene regulatory network analysis

Regulons were defined by a gene regulatory network called DoRothEA [62] containing a collection of TF—target gene interactions. decoupleR package was used to estimate regulon activities by a multivariate linear model (mlm) from the transcriptome data. Enrichment analysis of each regulon for HALLMARK and KEGG gene sets was performed by Fisher’s exact test utilizing clusterProfiler [63] package.

### Statistics

Statistical analyses were performed with Prism 7.0 (GraphPad Software, La Jolla, CA).

Statistical significance was determined by using unpaired *t* test for comparisons between two groups and one-way ANOVA for groups of 3 or more. All bar graphs represent the mean ± SEM, * denotes *p* < 0.05, ** denotes *p* < 0.005, *** denotes *p* < 0.0005, **** denotes *p* < 0.00005.

### Supplementary Information

Below is the link to the electronic supplementary material.

Increased Intracellular Calcium activates STAT3 in vitro. A) Live cell imaging of MCF10A cells expressing the RCaMP cytoplasmic calcium indicator in response to control media (top) and treatment with 10mM calcium + 1μM ionomycin. n= 20 cells for each of 3 experiments. B) Western analysis of total STAT3 and pSTAT3 from MCF10A cells under control and high calcium (10mM calcium + 1μM ionomycin). (n=3) C) Immunofluorescence for pSTAT3 in MCF10A cells under control or high calcium conditions (10mM calcium + 1μM ionomycin). D) Immunofluorescence for LAMP2 and Cathepsin B in MCF10A cells under control or high calcium conditions (10mM calcium + 1μM ionomycin). Scale bars represents 10μm. E) Lif, IL6, TGFβ3, CD14, and LBP mRNA expression in MCF10A cells under control or high calcium conditions (10mM calcium + 1μM ionomycin), as assessed by quantitative RT-PCR (QPCR) (n=3) Bar graphs represent the mean±SEM. ** denotes p<0.005, *** denotes p<0.0005, **** denotes p<0.00005 (TIF 42543 KB)

TFEB activation is not associated with calcineurin and mTOR signaling. A) Live cell Imaging of GCaMP3-TRPML1 and RCaMP in MCF10A cells grown at control (top) or high calcium (bottom) conditions. Red fluorescence is triggered by cytoplasmic calcium levels. Green fluorescence is triggered by calcium transport out of lysosomes through the TRPML1 calcium pump. B) Immunofluorescence for NFAT in MCF10A cells exposed to high calcium conditions (10mM calcium + 1μM ionomycin). Scale bars represents 10μm. C) Live cells stained by LysoTracker in MCF10A cells at control and high calcium conditions ± 1μM Cyclosporin A. D) Western blot analysis of pS6 (Ser235/236), S6, pS6K1, S6K1, pULK1 (Ser757), ULK1, and mTOR in MCF10A cells exposed to high calcium conditions (10mM calcium + 1μM ionomycin) (TIF 55785 KB)

Inhibition of cell cycle progression in early involution and associated with increased intracellular calcium. A) Hallmark and KEGG gene set enrichment plots of cell cycle related pathways from RNAseq results comparing day 10 lactation with day 2 involution in the mammary gland. B) Hallmark and KEGG gene set enrichment plots of cell cycle related pathways from RNAseq results comparing control and high calcium conditions (10mM calcium + 1μM ionomycin) in MCF10A cells (TIF 40504 KB)

Intracellular calcium level in day 10 lactating gland as assessed by GCaMP6f fluorescence. (Control). Z-stacks of multiphoton laser scanning microscopic images take from control lactating glands from BLG-Cre;Ai95 female mice. Blue fluorescence represents the second harmonic-generated signal from the collagen fibers within the fascia covering the glands. Green fluorescence is derived from increased intracellular calcium in MECs expressing the GCaMP6f calcium indicator (MP4 18626 KB)

Intracellular calcium level in mammary gland at 4 hours post teat-sealing as assessed by GCaMP6f fluorescence. Z-stacks of multiphoton laser scanning microscopic images take from 4 hours post teat-sealing from BLG-Cre;Ai95 female mice. Blue fluorescence represents the second harmonic-generated signal from the collagen fibers within the fascia covering the glands. Green fluorescence is derived from increased intracellular calcium in MECs expressing the GCaMP6f calcium indicator (MP4 14233 KB)

Intracellular calcium level in mammary gland at 8 hours post teat-sealing as assessed by GCaMP6f fluorescence. Z-stacks of multiphoton laser scanning microscopic images take from 8 hours post teat-sealing from BLG-Cre;Ai95 female mice. Blue fluorescence represents the second harmonic-generated signal from the collagen fibers within the fascia covering the glands. Green fluorescence is derived from increased intracellular calcium in MECs expressing the GCaMP6f calcium indicator (MP4 15952 KB)

Intracellular calcium level in mammary gland at 24 hours post teat-sealing as assessed by GCaMP6f fluorescence. Z-stacks of multiphoton laser scanning microscopic images take from 24 hours post teat-sealing from BLG-Cre;Ai95 female mice. Blue fluorescence represents the second harmonic-generated signal from the collagen fibers within the fascia covering the glands. Green fluorescence is derived from increased intracellular calcium in MECs expressing the GCaMP6f calcium indicator (MP4 10204 KB)

Intracellular calcium level in mammary gland at 24 hours post teat-sealing from BLG-Cre absence Ai95 female mice as assessed by GCaMP6f fluorescence. Z-stacks of multiphoton laser scanning microscopic images take from 24 hours post teat-sealing from Ai95 female mice (NO Cre). Blue fluorescence represents the second harmonic-generated signal from the collagen fibers within the fascia covering the glands. Green fluorescence is derived from intracellular calcium in MECs expressing the GCaMP6f calcium indicator (MP4 10217 KB)

Intracellular calcium level in mammary gland at 24 hours after pups were removed as assessed by GCaMP6f fluorescence. Z-stacks of multiphoton laser scanning microscopic images from 24 hours after pups removed at day 10 of lactation from BLG-Cre;Ai95 female mice. Blue fluorescence represents the second harmonic-generated signal from the collagen fibers within the fascia covering the glands. Green fluorescence is derived from increased intracellular calcium in MECs expressing the GCaMP6f calcium indicator (MP4 15582 KB)

Intracellular calcium level in mammary gland at 24 hours after pups were reintroduced following 24-hours without suckling as assessed by GCaMP6f fluorescence. Z-stacks of multiphoton laser scanning microscopic images from 24 hours after pups removed at 24 hours after pups reintroduced following 24-hours without suckling from BLG-Cre;Ai95 female mice. Blue fluorescence represents the second harmonic-generated signal from the collagen fibers within the fascia covering the glands. Green fluorescence is derived from increased intracellular calcium in MECs expressing the GCaMP6f calcium indicator (MP4 16857 KB)

## Data Availability

All data and information are included in the article and/or the supplemental information. RNA-seq data was deposited in NCBI’s GEO (Accession number GSE190031).

## References

[1] Baxter FO, Neoh K, Tevendale MC (2007). The beginning of the end: death signaling in early involution. J Mamm Gland Biol Neoplasia.

[2] Stein T, Salomonis N, Gusterson BA (2007). Mammary gland involution as a multi-step process. J Mamm Gland Biol Neoplasia.

[3] Watson CJ (2006). Involution: apoptosis and tissue remodelling that convert the mammary gland from milk factory to a quiescent organ. Breast Cancer Res.

[4] Watson CJ (2006). Post-lactational mammary gland regression: molecular basis and implications for breast cancer. Expert Rev Mol Med.

[5] Arnandis T (2012). Calpains mediate epithelial-cell death during mammary gland involution: mitochondria and lysosomal destabilization. Cell Death Differ.

[6] Hernandez LL, Collier JL, Vomachka AJ, Collier RJ, Horseman ND (2011). Suppression of lactation and acceleration of involution in the bovine mammary gland by a selective serotonin reuptake inhibitor. J Endocrinol.

[7] Jena MK, Jaswal S, Kumar S, Mohanty AK (2019). Molecular mechanism of mammary gland involution: an update. Dev Biol.

[8] Kreuzaler PA (2011). Stat3 controls lysosomal-mediated cell death in vivo. Nat Cell Biol.

[9] Kritikou EA (2003). A dual, non-redundant, role for LIF as a regulator of development and STAT3-mediated cell death in mammary gland. Development.

[10] Nguyen AV, Pollard JW (2000). Transforming growth factor beta3 induces cell death during the first stage of mammary gland involution. Development.

[11] Quarrie LH, Addey CV, Wilde CJ (1996). Programmed cell death during mammary tissue involution induced by weaning, litter removal, and milk stasis. J Cell Physiol.

[12] Stein T (2004). Involution of the mouse mammary gland is associated with an immune cascade and an acute-phase response, involving LBP, CD14 and STAT3. Breast Cancer Res.

[13] Zhao L (2004). Mammary gland remodeling depends on gp130 signaling through Stat3 and MAPK. J Biol Chem.

[14] Zhao L, Melenhorst JJ, Hennighausen L (2002). Loss of interleukin 6 results in delayed mammary gland involution: a possible role for mitogen-activated protein kinase and not signal transducer and activator of transcription 3. Mol Endocrinol.

[15] Lloyd-Lewis B (2018). Stat3-mediated alterations in lysosomal membrane protein composition. J Biol Chem.

[16] Sargeant TJ (2014). Stat3 controls cell death during mammary gland involution by regulating uptake of milk fat globules and lysosomal membrane permeabilization. Nat Cell Biol.

[17] Wang F, Gomez-Sintes R, Boya P (2018). Lysosomal membrane permeabilization and cell death. Traffic.

[18] Windelborn JA, Lipton P (2008). Lysosomal release of cathepsins causes ischemic damage in the rat hippocampal slice and depends on NMDA-mediated calcium influx, arachidonic acid metabolism, and free radical production. J Neurochem.

[19] VanHouten JN, Neville MC, Wysolmerski JJ (2007). The calcium-sensing receptor regulates plasma membrane calcium adenosine triphosphatase isoform 2 activity in mammary epithelial cells: a mechanism for calcium-regulated calcium transport into milk. Endocrinology.

[20] VanHouten J (2010). PMCA2 regulates apoptosis during mammary gland involution and predicts outcome in breast cancer. Proc Natl Acad Sci USA.

[21] Brini M (2009). Plasma membrane Ca(2+)-ATPase: from a housekeeping function to a versatile signaling role. Pflugers Arch.

[22] Brini M, Cali T, Ottolini D, Carafoli E (2013). The plasma membrane calcium pump in health and disease. FEBS J.

[23] Strehler EE, Zacharias DA (2001). Role of alternative splicing in generating isoform diversity among plasma membrane calcium pumps. Physiol Rev.

[24] Li M (1997). Mammary-derived signals activate programmed cell death during the first stage of mammary gland involution. Proc Natl Acad Sci USA.

[25] Chen TW (2013). Ultrasensitive fluorescent proteins for imaging neuronal activity. Nature.

[26] Madisen L (2015). Transgenic mice for intersectional targeting of neural sensors and effectors with high specificity and performance. Neuron.

[27] Selbert S (1998). Efficient BLG-Cre mediated gene deletion in the mammary gland. Transgen Res.

[28] Chapman RS (2000). The role of Stat3 in apoptosis and mammary gland involution. Conditional deletion of Stat3. Adv Exp Med Biol.

[29] Humphreys RC (2002). Deletion of Stat3 blocks mammary gland involution and extends functional competence of the secretory epithelium in the absence of lactogenic stimuli. Endocrinology.

[30] Tait L, Soule HD, Russo J (1990). Ultrastructural and immunocytochemical characterization of an immortalized human breast epithelial cell line, MCF-10. Cancer Res.

[31] Akerboom J (2013). Genetically encoded calcium indicators for multi-color neural activity imaging and combination with optogenetics. Front Mol Neurosci.

[32] Mahony R, Ahmed S, Diskin C, Stevenson NJ (2016). SOCS3 revisited: a broad regulator of disease, now ready for therapeutic use?. Cell Mol Life Sci.

[33] Sutherland KD, Lindeman GJ, Visvader JE (2007). Knocking off SOCS genes in the mammary gland. Cell Cycle.

[34] Sutherland KD (2006). c-myc as a mediator of accelerated apoptosis and involution in mammary glands lacking Socs3. EMBO J.

[35] Zhang L (2006). IL-6 signaling via the STAT3/SOCS3 pathway: functional analysis of the conserved STAT3 N-domain. Mol Cell Biochem.

[36] Carow B, Rottenberg ME (2014). SOCS3, a major regulator of infection and inflammation. Front Immunol.

[37] Lesina M (2011). Stat3/Socs3 activation by IL-6 transsignaling promotes progression of pancreatic intraepithelial neoplasia and development of pancreatic cancer. Cancer Cell.

[38] Napolitano G, Ballabio A (2016). TFEB at a glance. J Cell Sci.

[39] Raben N, Puertollano R (2016). TFEB and TFE3: linking lysosomes to cellular adaptation to stress. Annu Rev Cell Dev Biol.

[40] Acosta D (2016). LPA receptor activity is basal specific and coincident with early pregnancy and involution during mammary gland postnatal development. Sci Rep.

[41] Brady OA (2018). The transcription factors TFE3 and TFEB amplify p53 dependent transcriptional programs in response to DNA damage. Elife.

[42] Yin Q (2020). CDK4/6 regulate lysosome biogenesis through TFEB/TFE3. J Cell Biol.

[43] Medina DL (2015). Lysosomal calcium signalling regulates autophagy through calcineurin and TFEB. Nat Cell Biol.

[44] Park YJ, Yoo SA, Kim M, Kim WU (2020). The role of calcium-calcineurin-NFAT signaling pathway in health and autoimmune diseases. Front Immunol.

[45] Roczniak-Ferguson A (2012). The transcription factor TFEB links mTORC1 signaling to transcriptional control of lysosome homeostasis. Sci Signal.

[46] Napolitano G (2018). mTOR-dependent phosphorylation controls TFEB nuclear export. Nat Commun.

[47] Datto MB (1995). Transforming growth factor beta induces the cyclin-dependent kinase inhibitor p21 through a p53-independent mechanism. Proc Natl Acad Sci USA.

[48] Donovan J, Slingerland J (2000). Transforming growth factor-beta and breast cancer: cell cycle arrest by transforming growth factor-beta and its disruption in cancer. Breast Cancer Res.

[49] Polyak K (1994). p27Kip1, a cyclin-Cdk inhibitor, links transforming growth factor-beta and contact inhibition to cell cycle arrest. Genes Dev.

[50] Sitko JC (2008). SOCS3 regulates p21 expression and cell cycle arrest in response to DNA damage. Cell Signal.

[51] Khan MGM (2019). Hepatocyte growth control by SOCS1 and SOCS3. Cytokine.

[52] Jeong J (2021). MAL2 mediates the formation of stable HER2 signaling complexes within lipid raft-rich membrane protrusions in breast cancer cells. Cell Rep.

[53] Jeong J, Kim W, Kim LK, VanHouten J, Wysolmerski JJ (2017). HER2 signaling regulates HER2 localization and membrane retention. PLoS ONE.

[54] Sardiello M (2009). A gene network regulating lysosomal biogenesis and function. Science.

[55] Jeong J (2017). The scaffolding protein NHERF1 regulates the stability and activity of the tyrosine kinase HER2. J Biol Chem.

[56] Foley J (2001). Parathyroid hormone-related protein maintains mammary epithelial fate and triggers nipple skin differentiation during embryonic breast development. Development.

[57] Jeong J (2016). PMCA2 regulates HER2 protein kinase localization and signaling and promotes HER2-mediated breast cancer. Proc Natl Acad Sci USA.

[58] Kim D, Langmead B, Salzberg SL (2015). HISAT: a fast spliced aligner with low memory requirements. Nat Methods.

[59] Kovaka S (2019). Transcriptome assembly from long-read RNA-seq alignments with StringTie2. Genome Biol.

[60] Love MI, Huber W, Anders S (2014). Moderated estimation of fold change and dispersion for RNA-seq data with DESeq2. Genome Biol.

[61] Gu Z, Eils R, Schlesner M, Ishaque N (2018). EnrichedHeatmap: an R/Bioconductor package for comprehensive visualization of genomic signal associations. BMC Genom.

[62] Garcia-Alonso L, Holland CH, Ibrahim MM, Turei D, Saez-Rodriguez J (2019). Benchmark and integration of resources for the estimation of human transcription factor activities. Genome Res.

[63] Wu T (2021). clusterProfiler 40: a universal enrichment tool for interpreting omics data. Innovation (Camb).

