# Supplementary figures and images for: Intracellular calcium links milk stasis to lysosome-dependent cell death during early mammary gland involution
Source: Cell Mol Life Sci. 2024 Jan 12;81(1):29. doi: 10.1007/s00018-023-05044-8 (PMC10784359; doi:10.1007/s00018-023-05044-8)

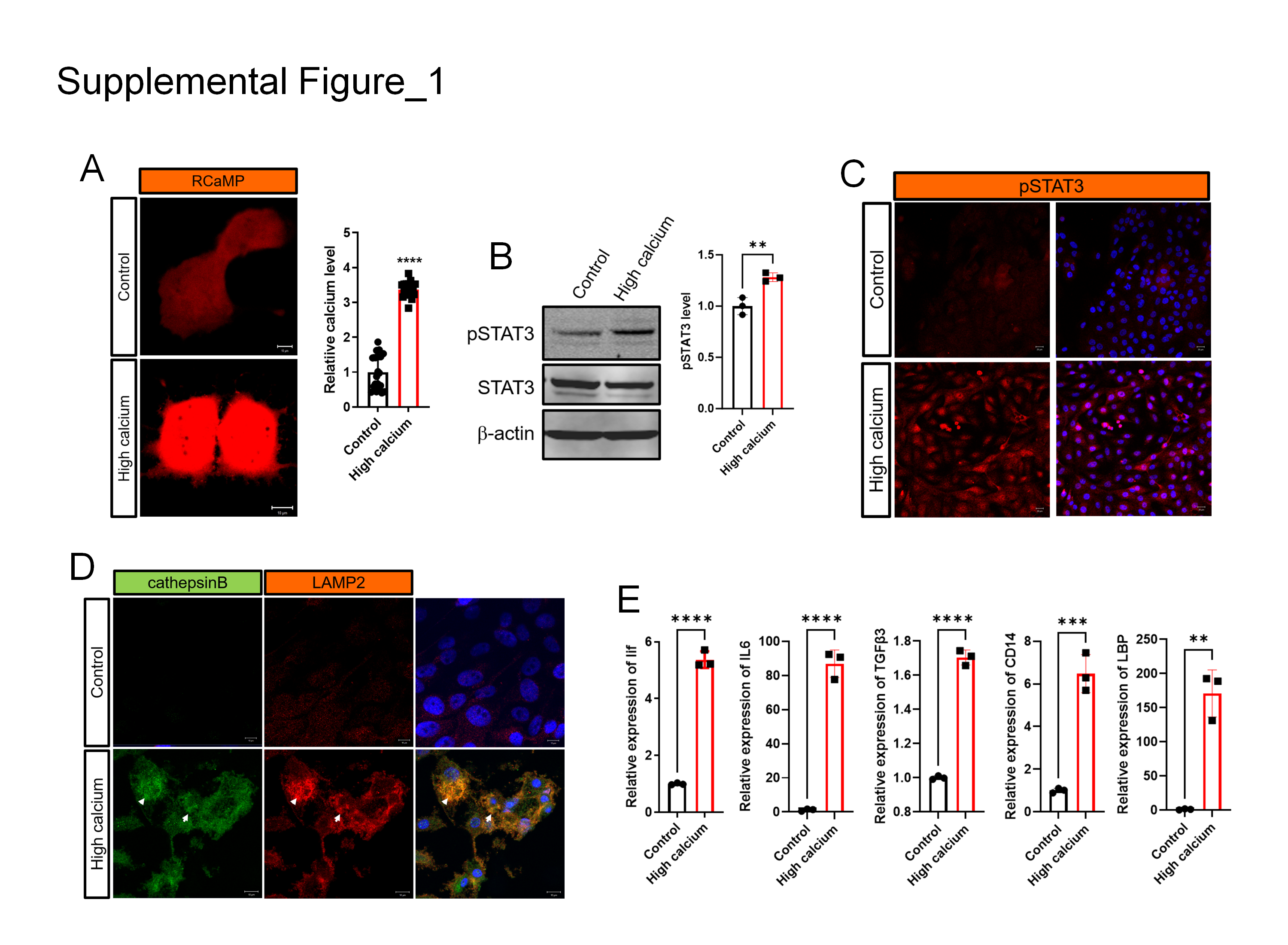

Supplement: Supplementary file 1 — Increased Intracellular Calcium activates STAT3 in vitro. A) Live cell imaging of MCF10A cells expressing the RCaMP cytoplasmic calcium indicator in response to control media (top) and treatment with 10mM calcium + 1μM ionomycin. n= 20 cells for each of 3 experiments. B) Western analysis of total STAT3 and pSTAT3 from MCF10A cells under control and high calcium (10mM calcium + 1μM ionomycin). (n=3) C) Immunofluorescence for pSTAT3 in MCF10A cells under control or high calcium conditions (10mM calcium + 1μM ionomycin). D) Immunofluorescence for LAMP2 and Cathepsin B in MCF10A cells under control or high calcium conditions (10mM calcium + 1μM ionomycin). Scale bars represents 10μm. E) Lif, IL6, TGFβ3, CD14, and LBP mRNA expression in MCF10A cells under control or high calcium conditions (10mM calcium + 1μM ionomycin), as assessed by quantitative RT-PCR (QPCR) (n=3) Bar graphs represent the mean±SEM. ** denotes p<0.005, *** denotes p<0.0005, **** denotes p<0.00005 (TIF 42543 KB) [file 18_2023_5044_MOESM1_ESM.tif]

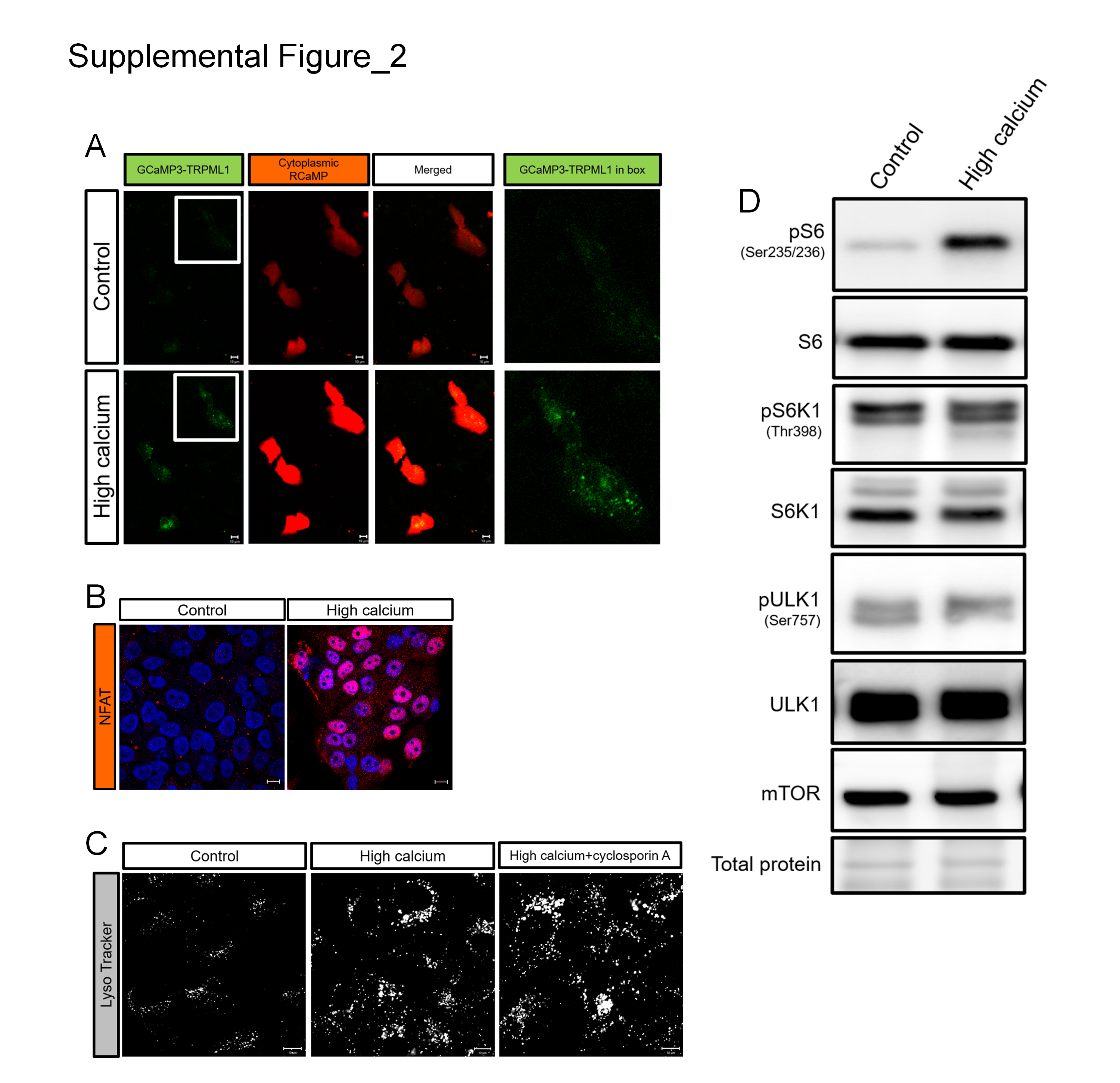

Supplement: Supplementary file 2 — TFEB activation is not associated with calcineurin and mTOR signaling. A) Live cell Imaging of GCaMP3-TRPML1 and RCaMP in MCF10A cells grown at control (top) or high calcium (bottom) conditions. Red fluorescence is triggered by cytoplasmic calcium levels. Green fluorescence is triggered by calcium transport out of lysosomes through the TRPML1 calcium pump. B) Immunofluorescence for NFAT in MCF10A cells exposed to high calcium conditions (10mM calcium + 1μM ionomycin). Scale bars represents 10μm. C) Live cells stained by LysoTracker in MCF10A cells at control and high calcium conditions ± 1μM Cyclosporin A. D) Western blot analysis of pS6 (Ser235/236), S6, pS6K1, S6K1, pULK1 (Ser757), ULK1, and mTOR in MCF10A cells exposed to high calcium conditions (10mM calcium + 1μM ionomycin) (TIF 55785 KB) [file 18_2023_5044_MOESM2_ESM.tif]

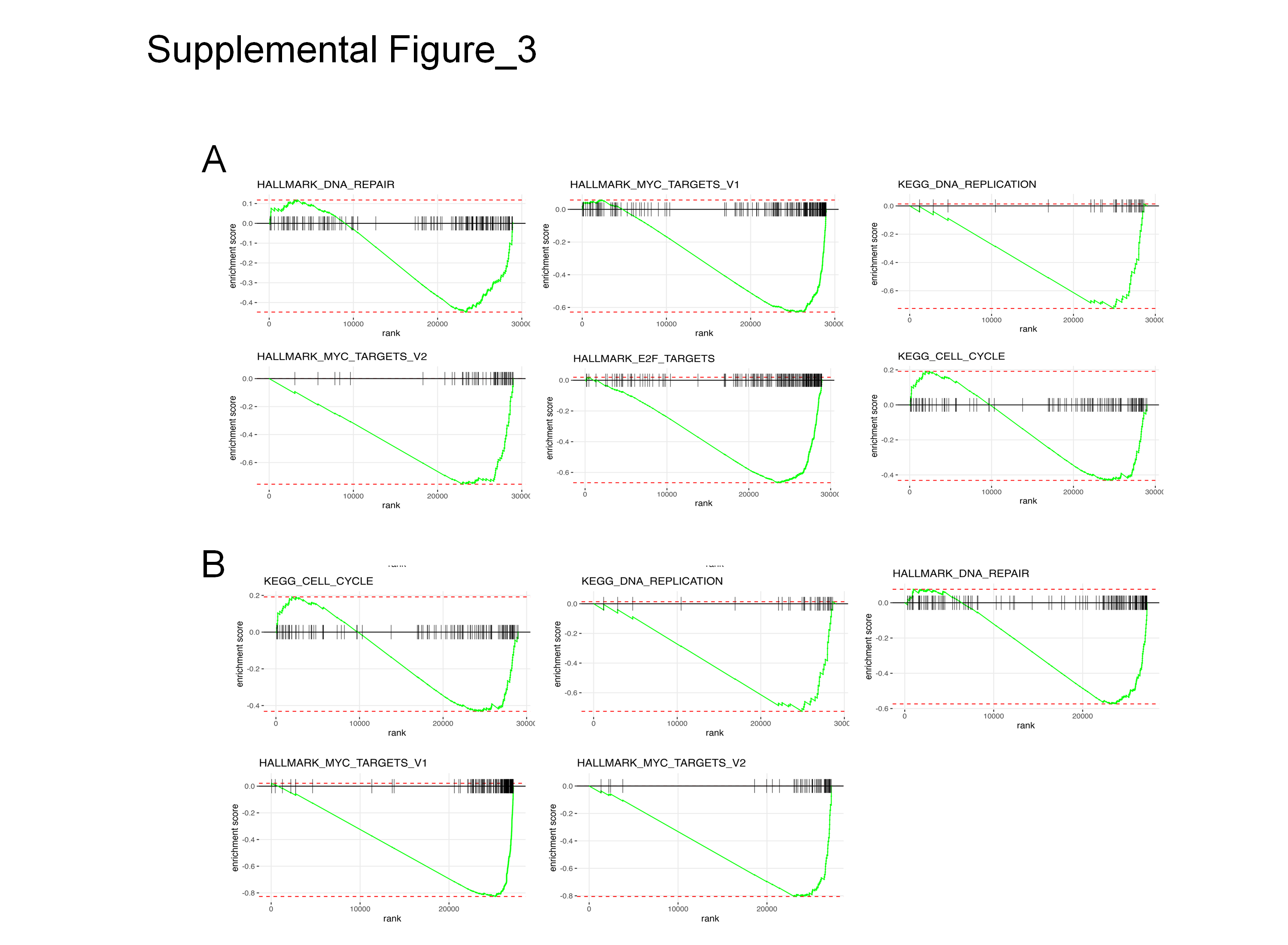

Supplement: Supplementary file 3 — Inhibition of cell cycle progression in early involution and associated with increased intracellular calcium. A) Hallmark and KEGG gene set enrichment plots of cell cycle related pathways from RNAseq results comparing day 10 lactation with day 2 involution in the mammary gland. B) Hallmark and KEGG gene set enrichment plots of cell cycle related pathways from RNAseq results comparing control and high calcium conditions (10mM calcium + 1μM ionomycin) in MCF10A cells (TIF 40504 KB) [file 18_2023_5044_MOESM3_ESM.tif]
